# Human Biomonitoring Guidance Values for Deoxynivalenol Derived under the European Human Biomonitoring Initiative (HBM4EU)

**DOI:** 10.3390/toxins16030139

**Published:** 2024-03-07

**Authors:** Marcel J. B. Mengelers, Annick D. van den Brand, Shensheng Zhao, Rudolf Hoogenveen, Eva Ougier

**Affiliations:** 1 National Institute for Public Health and the Environment (RIVM), 3720 BA Bilthoven, The Netherlandsrudolf.hoogenveen@rivm.nl (R.H.); 2 French Agency for Food, Environmental and Occupational Health & Safety (Anses), 94701 Maisons-Alfort, France

**Keywords:** deoxynivalenol, mycotoxin, human biomonitoring guidance value, HBM-GV, HBM4EU

## Abstract

The mycotoxin deoxynivalenol (DON) was one of the priority substances in the European Joint Human Biomonitoring Initiative (HBM4EU) project. In this study, to better interpret the actual internal exposure of DON in the general population and safeguard public health, human biomonitoring guidance values of DON for the general population (HBM-GV_GenPop_) were derived. The HBM-GV_GenPop_ of DON was based on either the total DON (DON and its glucuronides) or DON’s main metabolite (DON-15-GlcA) levels in 24-h urine samples, resulting in a HBM-GV_GenPop_ of 0.023 µg/mL for the total DON or a HBM-GV_GenPop_ of 0.020 µg/mL for DON-15-GlcA. The use of 24-h urine samples is recommended based on the fact that DON and its metabolites have a short elimination half-life (T_1/2_), and 95% of the cumulative amount was excreted within 12 h after DON intake. The T_1/2_ for DON, DON-15-GlcA, and total DON were estimated to be 2.55 h, 2.95 h, and 2.95 h, respectively. Therefore, a 24-h urine sample reflects almost all of the DON exposure from the previous day, and this type of sample was considered for the derivation of a HBM-GV_GenPop_ for DON.

## 1. Introduction

The European Joint Human Biomonitoring Initiative (HBM4EU) was a joint effort of 30 countries and the European Commission, aiming to coordinate and advance human biomonitoring (HBM) in Europe and support policy development [1]. Within the framework of the HBM4EU, human biomonitoring guidance values (HBM-GVs) were derived for several HBM4EU priority substances [2]. A systematic and transparent methodology to derive HBM-GVs was proposed within HBM4EU, which was described by Apel et al. [3].

One of the HBM4EU’s priority substances was the mycotoxin deoxynivalenol (DON) [4]. DON can co-occur in grains, cereal-based food, and feed together with its acetyl derivatives (3-acetyl-DON (3-Ac-DON) and 15-acetyl-DON (15-Ac-DON)) and modified forms (e.g., DON-3-glucoside (DON-3G)). Exposure to such acetylated and modified forms can add to the exposure of DON, considering that they are converted back to DON in the gastrointestinal tract. Since these compounds are not (yet) always included in routine food and feed analyses, the total exposure to DON can be best assessed via HBM. Within the HBM4EU, new HBM data were generated within aligned studies, and previously published HBM data on DON were gathered [5,6,7]. For risk assessment purposes, there is a need to derive a HBM-GV for DON to assess the risk of the DON exposure estimated in HBM studies.

DON is a mycotoxin mainly produced by *Fusarium graminearum* and *Fusarium culmorum.* These fungal species can infest (raw) agricultural commodities and thereby introduce mycotoxins into the food chain. Following oral exposure, DON can be rapidly absorbed by humans and most animals [8,9,10,11], and is widely distributed to different organs [9,10]. In the liver, DON can be partly de-epoxidized to the less toxic phase I metabolite DOM-1 (de-epoxy-DON). Subsequently, the formed DOM-1 might be further conjugated (via glucuronidation and sulfation) into different phase II metabolites. In humans, the glucuronidation of DON is considered to be the main phase II metabolic pathway resulting in the formation of different metabolites, such as DON-15-glucuronide (DON-15-GlcA) and DON-3-glucuronide (DON-3-GlcA) [9,12,13]. Urinary excretion is the main excretion route of DON following oral exposure, as approximately 70% of ingested DON is excreted via urine as DON and its glucuronides and only a low percentage of ingested DON was found in faeces [13,14,15]. Based on a human intervention study described by Vidal et al. [14] and Mengelers et al. [11], DON and its glucuronides are rapidly excreted via the urine, as 95% of the cumulative excreted amount of DON was already excreted after approximately 12 h. This was also observed in another study by Van den Brand et al. [16], which modelled the urinary excretion of DON in Norwegian volunteers from the EuroMix HBM study. In addition, approximately 72% of the total excreted DON was excreted as its glucuronidated forms within 24 h, with the ratio of the cumulative amounts (in nmol) of DON-15-GlcA being 2.6-fold higher than that of DON, and DON-15-GlcA being 4.5-fold higher than that of DON-3-GlcA [11,14].

DON is also known as vomitoxin, and acute DON toxicosis has been associated with symptoms such as nausea, vomiting, diarrhoea, abdominal pain, headaches, dizziness, fever, and in severe cases, bloody stool in humans [9]. A NOAEL of 26 µg DON/kg bw for a single eating occasion was established by the European Food Safety Authority (EFSA) Panel on Contaminants in the Food Chain (CONTAM Panel), resulting in a group acute reference dose (ARfD) of 8 µg/kg bw per eating occasion for the sum of DON, 3-Ac-DON, 15-Ac-DON, and DON-3G [9]. Given that there is no available data on the chronic effects of DON exposure in humans, the EFSA CONTAM Panel identified reduced body weight gain in a long-term experimental study with mice as the most relevant and suitable critical chronic effect for human risk assessment [9,17]. From this study, a benchmark dose lower confidence limit for a 5% response (BMDL05) of 0.11 mg/kg bw per day was derived from a dose–response curve between the oral exposure to DON and reduced body weight gain in mice [9]. By further applying the default uncertainty factor of 100 for inter- and intra-species variability, a so-called group tolerable daily intake (TDI) of 1 µg/kg bw/day was established for the chronic risk of the sum of DON, 3-Ac-DON, 15-Ac-DON, and DON-3G.

In the present study, the group-TDI was used to derive a HBM-GV for DON in urine for the general population. Occupational exposure to DON via inhalation during tasks involving close contact with organic dust or mould, such as storing, loading, handling, or processing contaminated materials (grain, waste, and feed) can occur, as well as through the handling of animals in animal production settings due to activities related to DON-contaminated litter [18,19]. Dermal absorption may also take place through the deposition of mycotoxin-containing dust particles on the skin or through contact with work surfaces contaminated with such dust particles [20,21]. However, due to the lack of toxicokinetic data for the inhalation and dermal routes of exposure, no HBM-GVs for occupationally exposed adults to DON could be derived.

## 2. Results and Discussion

### 2.1. Selection of a DON Biomarker of Exposure (Total (Deglucuronidated) DON or DON-15-Glucuronide)

In humans, the majority of ingested DON is excreted as glucuronides [13,14,22], withDON-15-GlcA as the major urinary biomarker of DON exposure [12,16,23,24,25]. This is consistent with observations from various epidemiological studies [9,26]. However, the ratio of glucuronidated metabolites to total excreted DON also appears to vary between sexes and age groups [26,27,28,29,30,31,32]. Therefore, within a HBM4EU interlaboratory study, the total urinary DON (free DON, deglucuronidated DON-15-GlcA, and DON-3-GlcA) was selected as the preferred biomarker of DON exposure. A requirement for using the total DON as a biomarker is that the urine samples are quantitatively deglucuronidated via enzymatic treatment. The optimal laboratory conditions to achieve complete de-glucuronidation are described in the HBM4EU interlaboratory comparison report [33]. Therefore, we first derived a HBM-GV_GenPop_ based on the total DON levels in the urine. 

However, since DON-15-GlcA is the major urinary biomarker for DON exposure, we also derived a HBM-GV_GenPop_ based on the DON-15-GlcA levels in the urine. Given that no enzymatic deglucuronidation is necessary to analyse this metabolite, no additional uncertainty was introduced in the assessment. However, the practical use of a HBM-GV using the measured DON-15-GlcA in the urine is often hampered by the fact that (1) the total fraction of DON-15-GlcA in the total excretion of DON may vary between different individuals [31,34,35], and (2) an appropriate analytical standard material to analyse DON-15-GlcA, although nowadays commercially available, has not been qualified yet as a reference material.

### 2.2. Derivation of the HBM-GV for DON

#### 2.2.1. Approach for Deriving the HBM-GV

Given that no human studies are available to derive a HBM-GV for the general population, we estimated the HBM-GV for DON using the second approach described in Apel et al. [3], in which the HBM-GV was translated from a selected external toxicity reference value (TRV). In this case, the TRV consisted of the group-TDI for the sum of DON and its acetylated and modified forms derived by EFSA [9]. The derivation of the HBM-GV for DON for the general population is provided in Section 4 of the Materials and Methods.

A pre-requisite of “translating” a TRV to a HBM-GV, according to Apel et al. [3], is a steady-state condition. Given the short elimination half-life (T_1/2_) of DON (and its glucuronides) of approximately 3 to 4 h and the average exposure interval (between meals), this condition is (usually) not met. Based on the data from human intervention studies described by Vidal et al. and Mengelers et al. [11,14], the T_1/2_ of DON, DON-15-GlcA, and the total DON were estimated to be 2.55 h, 2.95 h, and 2.95 h, respectively, as reported in part VIII of deliverable 5.9 of the HBM4EU [36]. The T_1/2_ of the total DON is driven by the larger contribution of the T_1/2_ of DON-15-GlcA, since this is the major biomarker of DON in urine. A similar elimination T_1/2_ (2.9–3.6 h) was reported by Faeste et al. [10], which was based on the predicted plasma clearance (based on in vitro data) and distribution volume (based on allometric scaling). A study that modelled the renal excretion of DON in humans in an everyday situation estimated a T_1/2_ of 4.0 h [16].

As a result of this short T_1/2_, 95% of the cumulative amount excreted (P95) of all consumed DON is excreted within 12 h [11,16]. Therefore, when collecting a 24-h urine sample that includes the collection of a first morning urine void at the end of the 24-h period, almost all consumed DON in that 24-h period is captured in the urine sample. Therefore, the mass balance equation as described in option two for deriving HBM-GVs by Apel et al. [3] can be used to derive a HBM-GV_GenPop_ for DON. However, the use of this equation normally assumes steady-state conditions, which in the present case is not met, considering DON’s short T_1/2_. Nonetheless, it is applicable to the specific situation whereby 95% of the total DON is excreted within 12 h, and the total DON will be excreted almost completely after the last dosing (i.e., the evening meal).

#### 2.2.2. Parameters Used for the Derivation of the HBM-GV 

To calculate the HBM-GVs for the adult general population based on either the total DON or DON-15-GlcA levels in the urine, three parameters were required in total, namely, the group-TDI, the urinary excretion factors (Fues) and the adult 24-h urinary flow rate.

In the present study, the reported group-TDI for DON of 1 µg/kg bw/day [9] was used for the HBM-GV_GenPop_ derivations. The first HBM-GV_GenPop_ was based on the total DON levels in the urine, while the second HBM-GV_GenPop_ was based on the DON-15-GlcA levels in the urine. Given that the recently derived group-TDI by EFSA [9] was used, no further attempt was performed on the identification and evaluation of another critical effect of DON in the present study. However, a literature search was performed to gather the human studies investigating the effect of DON since the release of EFSA’s scientific opinion; however, this did not result in the identification of new studies. 

Mengelers et al. [11] calculated and reported a so-called reversed dosimetry factor (RDF) for the total DON and DON-15-GlcA, which were 1.45 and 2.67, respectively. The RDF is the ratio of DON intake to the calculated cumulative excreted amount, and it is the inverse of the Fue. As the distribution of the ratios is skewed, a logistic transformation was applied to calculate the confidence bounds. The Fue for the total DON was estimated to be 0.69 with a 95% confidence interval (95% CI) of 0.14–0.97, whereas the Fue for DON-15-GlcA was estimated to be 0.37 with a 95% CI of 0.13–0.71 (also see Table A1). These Fues were subsequently used to derive a HBM-GV_GenPop_ for the total DON and DON-15-GlcA (Section 4.1.2). The Fues for the total DON and DON-15-GlcA obtained in this study were very similar to the excretion and glucuronidation profile of another human intervention study by Warth [13]. In this study, the DON intake was analysed rather than estimated, resulting in a higher level of certainty in the Fue. Note that the Fue values above were estimated for adults because, to the best of our knowledge, no Fue values specific to children have been derived based on 24-h urine samples. In addition, contradicting results on the extent of glucuronidation in children have been published. It appears that the ratio of free DON to the total DON (varying between 11 and 25%) is slightly higher in children compared to adults, as reported by several authors [25,30,31,32,35]. Yet, in contrast to the aforementioned studies, Warensjö Lemming [34] stated that the percentage of free DON to total DON (defined as the free DON+DON-15-GlcA) was much higher in Swedish adolescents, with approximately 60% free DON.

The other parameter required to derive the HBM-GVs is the average urinary flow rate. In the present study, an average 24-h urinary flow rate in adults of 29.4 mL/kg bw/day was used, based on the data from the study by Mengelers et al. [11]. This value was calculated based on the average 24-h urine volume of the 20 volunteers (mean age: 32 years, range: 18–61 years, 11 women and 9 men) and their respective body weights, hence it represents the value of an adult. It was found to be higher than the urinary flow rate of 20 mL/kg bw/day proposed by the German HBM Commission [3]. But interestingly, an increase in 24-h urinary flow rates was found for the urine samples collected from 1997 to 2016 in young adults according to Lermen, et al. [37]. This justifies our deviation from the default urinary flow rate usually used for HBM-GV derivation for adults and indicates that the default daily urinary flow rate may need to be revised accordingly [3]. Apart from that, no significant differences between the male and female 24-h urinary flow rates were reported [3,38], although a small difference was observed between males and females when the 24-h urinary flow rate was calculated based on the data from a human intervention study [11,14]. Since the measured daily urinary flow rate has a significant impact on the result of a HBM-GV, caution is required when selecting a daily urinary flow rate for a certain target group (if appropriate Fue values are available). Especially for children, a lower urinary daily volume was reported by Apel, et al. [3] and Warensjö Lemming et al. [34].

#### 2.2.3. Calculation of the HBM-GV for the General Adult Population

Using the parameters described in the previous section, we derived a HBM-GV_GenPop_ of 0.023 µg/mL for the total DON and a HBM-GV_GenPop_ of 0.020 µg/mL for DON-15-GlcA by translating the DON group-TDI value derived by EFSA into “internal” biomonitoring concentrations using a mass balance equation with the specific Fue values (Section 4.1.3).

However, to date, few epidemiological studies using DON internal exposure have used 24-h urine samples, while quite some studies have used first-morning urine samples to analyse the DON levels. Comparing the measured DON concentrations in first-morning urine samples to the group-TDI led to different challenges, including the rapid elimination of DON and the intake of DON at different time points during the day preceding sampling. The only scenario in which a HBM-GV could be derived for the DON concentrations in morning urine samples assumes a constraint scenario with three conditions: (1) a first-morning urine sample must have been collected before the first DON exposure of that day (i.e., before breakfast); (2) the last DON exposure during the preceding day must have occurred at or around the evening meal (otherwise, the total DON from the last exposure moment would not be completely excreted in the morning urine); and (3) no urine must have been voided during the period between the last evening meal and the first-morning urine (approximately 12 h). Provided that these three conditions are met, practically all the DON intake during an evening meal will be excreted in the morning urine sample. For example, if the last DON intake was at the level of the group- TDI (i.e., 1 µg/kg bw/day) and taking the Fue for the total DON into account, the excreted amount in the morning urine would be 0.69 µg/kg bw. Using an average 12-h urinary flow rate of 14.7 mL/kg bw (based on the average 24-h urinary flow rate), a HBM-GV_GenPop_ of 0.047 µg/mL (=0.69/14.7) for the total DON in the first-morning urine sample could be considered. However, it is unlikely in an every-day situation that no urine is voided between the evening meal and the first morning void (third condition). Therefore, a level above 0.047 µg/mL (rounded value) in a first-morning urine sample would indicate that the previous day, the group TDI has been exceeded. However, more importantly, one cannot conclude that a total DON level in a first-morning urine sample below 0.047 µg/mL would indicate that the group-TDI was not exceeded the previous day. 

In other words, a HBM-GV for interpreting the DON levels in a morning urine sample is constrained because of the short T½ of DON. It is, therefore, questionable as to how indicative a morning urine sample would be to estimate the daily DON exposure (for the reasons as indicated in the above paragraph). However, on a population basis, this discussion appears less relevant as the use of urine samples collected at a time point during the day or 24-h urine samples produces comparable results if the population is large enough and the sampling is random [39]. Using a large population to estimate the daily DON exposure will, however, be accompanied by large sampling and analytical costs. 

**Figure 1:**
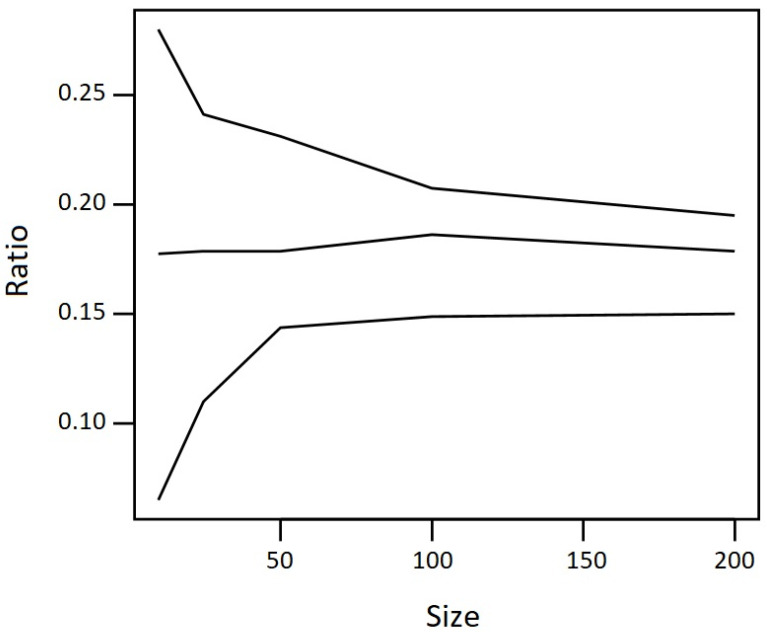
Simulation of the fraction of first-morning urine: 24-h urine (ratio on the *y*-axis), depending on the size of the population (size on the *x*-axis). The median ratio is reflected by the middle line surrounded by the 95% confidence interval.

To better understand the effect of increasing one’s study population to reduce the variation between morning urine samples and 24-h urine samples, the data from Van den Brand et al. [16] was re-analysed. Using Monte Carlo iterations, increasing population sizes were simulated from the distribution of the ratio of DON-15-GlcA in the morning urine samples and 24-h urine samples from the 49 volunteers in that study (Figure 1). One hundred distributions were generated per simulated population size. The effect of the population size on the median ratio appears small. The uncertainty around this median ratio is affected by the population size, up to a population size of approximately 100 individuals. 

Based on Figure 1, it can be concluded that the confidence interval (CI) of the median fraction of DON-15-GlcA in the morning urine as compared to the 24-h urine decreases when the population size increases to approximately 100 individuals. Above this population size (*n* > 100), the CI does not appear to further decrease. 

### 2.3. Level of Confidence Attributed to the HBM-GV 

A high, medium, or low level of confidence (LoC) is attributed to each derived HBM-GV according to the HBM-GV derivation strategy [3]. This LoC aims to reflect the uncertainties related to the derived value. It is determined from the confidence level assigned by expert judgment to several single parameters described hereafter.

#### 2.3.1. Nature and Quality of the Epidemiological/Toxicological Data and Toxicokinetic Data

DON is also known as vomitoxin due to its ability to induce acute vomiting in animals after high dosing. Many incidents of human intoxication have been associated with DON exposure, and its acute emetic effect is similar to that in animals. A group-ARfD of 8 µg/kg bw based on human data of vomiting and gastrointestinal effects was set by EFSA [9]. As human data on chronic high exposure to DON are lacking, a group-TDI was established by EFSA [9] based on the reduced body weight gain in mice. The fact that human data are lacking to assess the chronic risks for humans points towards a low LoC for this parameter.

However, two quantitative studies on DON urinary excretion after oral exposure informing the toxicokinetic properties of DON in humans are available [11,14]. These studies included a sufficient number of subjects, both men and women.

A medium level of confidence (LoC) was assigned to this aspect.

#### 2.3.2. The Critical Effect and the Mode of Action

The group-TDI derived by EFSA [9] for the sum of DON and its acetylated and modified forms was used in the present study and “translated” into an internal HBM-GV_GenPop_. Based on EFSA [9], the critical effect used for the derivation of the group-TDI was reduced body weight gain in mice, the most common effect of long-term dietary exposure in animals besides anorexia. Feed refusal and reduced feed intake have been associated with hormonal and immunotoxic effects of DON, since changes in satiety hormones and changes in proinflammatory cytokines have been linked to DON-induced anorexia [9]. However, the available data was too weak for EFSA to identify any critical endpoint based on immunotoxic or hormonal effects in experimental animals related to chronic DON exposure. EFSA also stated that DON is genotoxic in vitro but that the data available on the genotoxicity of DON in vivo were inconclusive [9]. A literature search was performed at the end of 2019 within the HBM4EU project to identify relevant human health effects of DON for the development of an adverse outcome pathway [40]. The review did not identify any human studies on DON, or any new toxicological studies published after the literature search conducted by EFSA that would possibly indicate another critical chronic effect other than reduced body weight gain for human risk assessment.

A medium LoC was attributed to this aspect, as uncertainties existed regarding the effects that would appear at a lower dose than the retained critical effect.

#### 2.3.3. The Selection of the Key Study Regarding the Critical Effect

The key study selected by EFSA to derive the group-TDI was a 2-year feeding study in mice by Iverson et al. [17]. This study was designed to examine the effect of DON exposure on carcinogenicity, but the data on the general toxicity of the animals were also reported. The design of the study was very similar to the OECD Guidelines 452: Chronic Toxicity Studies (2009). It was considered a good-quality experimental study.

A high LoC was attributed to this aspect. 

#### 2.3.4. The Selection of the POD

In the present study, no point of departure (POD) was selected by the authors since the HBM-GV_GenPop_ was derived based on the group-TDI reported by EFSA [9]. 

Based on the EFSA [9], the POD was a BMDL05 of 0.11 mg/kg bw/day for reduced body weight gain based on the body weight data generated by Iverson et al. [17] for female and male mice combined. The BMD approach for continuous data was applied using the default BMR of 5% in the absence of statistical or toxicological considerations supporting a deviation. 

A high LoC was attributed to this aspect. 

#### 2.3.5. The Selection of the Other Required Parameters

The study that was selected to derive the Fue was a human intervention study by Mengelers et al. [11]. In this study, 16 out of 20 adult volunteers (mean age: 32 years, range: 18–61 years, 11 women and 9 men) were administered 1 μg/kg bw of DON or DON-3G via oral bolus after consuming a cereal-free diet for 3 days [14]. Four volunteers served as the control group. After the administration of the bolus, every urine sample over a 24-h period was collected and analysed for DON and its metabolites. The authors calculated a RDF for the individual metabolites in the urine as well as for the total fraction of DON recovered in the urine. Considering that the bolus that the subjects received was the same as the group-TDI value, the total recovered fraction of DON in the urine as well as the fraction of DON-15-GlcA could confidently be used for the purpose of deriving a HBM-GV_GenPop_ from the group-TDI. It is important to note that the authors also reported the CI around the RDFs, which describes the variation in the excretion between the subjects.

To derive the HBM-GV_GenPop_, the urinary flow rate of an adult was required. This value was also calculated from the human intervention study by Mengelers et al. [11], which was 29.4 mL/kg bw/day. However, a different default urinary flow rate for adults (20 mL/kg bw/day) was proposed by the German HBM commission. The use of the urinary flow rate from the German HBM Commission resulted in a higher HMB-GV than the HMB-GV calculated based on the data from Mengelers et al. [11]. Therefore, caution must be taken when selecting the urinary flow rate. If no strong arguments can be provided in the case of multiple available flow rates, one can select the highest urinary flow rate, as this would result in a conservative HBM-GV.

A high LoC was attributed to this aspect. 

#### 2.3.6. The Extrapolations across and within Species

In the absence of specific data, a default uncertainty factor of 10 was applied to take into account the inter- and intra-species differences in the group-TDI derivation by EFSA. 

A low LoC was attributed to this aspect.

#### 2.3.7. Overall LoC

Considering the sum of the LoCs attributed to the single parameters, the overall LoC assigned to the HBM-GVs derived for DON based on 24-h urine samples was medium.

### 2.4. Limitations and Uncertainties of the Proposed HBM-GV

The parameters used to derive the HBM-GV for the general population were based on a human intervention study where the urinary excretion after a known exposure to DON was followed. Although the study design eliminated the uncertainties on DON exposure, variation in the excretion of DON was observed (and was included in the HBM-GV). Future studies or a meta-analysis could be conducted to reduce the uncertainty around the critical parameters, such as the Fue of DON-15-GlcA and the ratio of DON-15-GlcA to the total DON in addition to further research on the 24-h urinary flow rate. The currently derived HBM-GV applies to adults and was not derived for children. There have been conflicting data published on the glucuronidation profile in children. Future studies may focus on the excretion and glucuronidation ratio of DON and metabolites in children. Deriving this information, in addition to updated information on the 24-h flow rate in children may result in an estimation of a HBM-GV for children.

Limited research has been published with respect to ethnic differences in the excretion and glucuronidation profile of DON. There is currently no indication that this differs between ethnic groups, at least not in Chinese adult volunteers [30]. Rather, other factors like smoking, obesity, and gender are known to influence glucuronidation [41]. Nonetheless, research may be dedicated on the human biomonitoring of glucuronidation profiles in ethnically diverse populations and this information should be included as a parameter in HBM population studies to reveal any possible differences between ethnicities. 

We have also derived a HBM-GV based on the single metabolite DON-15-GlcA. Various studies have reported similar excretion percentages of DON-15-GlcA over 24 h and the single use of DON-15-GlcA is also practically appropriate as shown by Van den Brand et al. [16]. We acknowledge, however, that the uncertainty is lower when using the total DON for biomonitoring purposes (measured as DON-15-GlcA, DON-3-GlcA, and free DON), because the variability in the metabolite excretion ratio between individuals is disregarded. Yet, in practice, we have observed that in the study by Van den Brand et al. [16], DON-15-GlcA was indeed identified as DON’s major metabolite but in the majority of the volunteers’ samples DON-3-GlcA or free DON could not be detected. Thus, the individual DON metabolites can only be detected in spot urine samples whenever the method’s limits of detection are sufficient, which may lead to practical difficulties. 

## 3. Conclusions

This is the first time that HBM-GVs of DON for the general population were derived based on urinary biomarkers (either the total DON or DON-15-GlcA) quantified over a collection period of 24 h. The use of biomarker levels in a 24-h urine sample to derive these guidance values is considered adequate as it was able to capture almost all of the daily DON exposure and reflects the actual internal exposure of DON in the general population. Therefore, these newly derived HBM-GVs can be used for the risk assessment of DON and may better safeguard public health. 

## 4. Materials and Methods

### 4.1. Derivation of the HBM-GV for DON

#### 4.1.1. Approach for Deriving the HBM-GV

According to Apel et al. [3], three different approaches could be applied to derive HBM-GVs for the general population. The first approach is to derive the HBM-GV based on a relationship between a selected critical effect (i.e., the most sensitive adverse health effect) and the selected biomarker(s) as observed in humans. The second approach is to estimate the biomarker(s) level(s) that corresponds to a selected external TRV proposed by European or international bodies (e.g., the TDI or acceptable daily intake (ADI)). The third approach is to derive the HBM-GV based on a critical effect and a critical dose reported in animal toxicological studies. If available and reliable, human data are preferred over animal data. 

Within the HBM4EU project, a literature search was performed to identify the relevant human health effects of DON for the development of an adverse outcome pathway. The review did not identify any new human studies on DON or any new toxicological studies that would indicate another critical chronic effect other than reduced body weight gain for human risk assessment. These findings have been published in a manuscript by Van den Brand et al. [40]. Given that no human studies are available to derive a HBM-GV for the general population, the second approach was selected.

#### 4.1.2. Parameters Used for the Derivation of the HBM-GV (Three Parameters)

Based on the equation presented below, in total, three parameters were needed for the calculation of the HBM-GV_GenPop_, these were: (1) the group-TDI, (2) Fues, and (3) 24-h urinary flow rate. In the present study, the group-TDI of 1 µg/kg bw/day that was derived based on the reduced body weight gain in mice by EFSA [9] was used due to the absence of human data. For the Fues, the data obtained from Mengelers et al. [11] were used, with a Fue of 0.69 for the total DON and 0.37 for DON-15-GlcA. The body weight-adjusted urinary flow rate for adults was also obtained using the human volunteer data from Mengelers et al. [11], which was 29.4 mL/kg bw/day. 

#### 4.1.3. Derivation of the HBM-GV for the General Population (Equation)

In the present study, the HBM-GV_GenPop_ for DON was derived based on the second approach as described by Apel et al. [3] (see above). However, the prerequisite of using this approach is that the compound should be in a steady-state condition [3]. This condition is not met in the case of DON, because the elimination T_1/2_ of the total DON and DON-15-GlcA are shorter than the dosing interval (when assuming DON exposure at a total of three times per day: breakfast, lunch, and evening meals). Therefore, a steady state would not be reached even after continuous (daily) exposure (Section 2.2.1). Yet, the time it takes to reach 95% of the cumulative amount excreted of ingested DON is 12 h [11,16], indicating that the period of urine collection used in the present study (24 h) is able to capture almost all of the daily DON exposure. Thus, the HBM-GV_GenPop_ for DON could be derived based on the same equation as that in the second approach (see below).

The equation used to derive the HBM-GV_GenPop_ for DON, which is based on the level of the selected biomarker of exposure (urinary total DON or DON-15-GlcA) in a 24-h urine sample, is shown below:

$$HBM\text{-}{GV}_{GenPop}=\frac{group\;TDI\times Fue}{24\text{-}h\;urinary\;flow\;rate\;adjusted\;to\;the\;body\;weight}$$

### 4.2. Level of Confidence (Six Factors)

In the present study, the possible uncertainties related to six impact factors were discussed. These impact factors were assessed to assign an overall LoC to the derived HBM-GVs, which were: (1) the nature and quality of the epidemiological/toxicological data (including the toxicokinetic data); (2) the data regarding the critical effect and the mode of action; (3) the selection of the key study; (4) the selection of the POD; (5) the selection of the other required parameters; and (6) the extrapolations across and within species.

Three levels of confidence were distinguished: low, medium, and high. These levels were established principles described by Apel et al. [3], but they also rely on expert judgement. The LoC does not directly relate to the level of protection towards adverse effects conferred by the HBM-GV, as a value with a low LoC may have been derived with very conservative default assumptions. They however help to point out the research required to reduce the uncertainties.

## Data Availability

Data are contained within the article.

## Appendix A

**Table A1:** Overview (factsheet) of all the parameters relevant for the derivation of a HBM-GV of DON for the general population.

Deoxynivalenol	Target Population: General Population	
Parameter	Value/Descriptor	Comments
HBM-GV unit	µg/L	
HBM-GV year of issue	2021	
CLP-INDEX-Nr.	-	
EC-Nr.	610-668-0	
CAS-Nr.	51481-10-8	
Harmonized CLP classification	-	
Molar mass	296.31 g/mol	
Identification	Biomarker(s) of exposure	
Molar mass of biomarker(s)	DON: 296.31 g/mol DON-15-Glucuronide: 472.4 g/mol	
Half-life of selected biomarker(s) *	DON: 2.55 h (CI: 2.2–2.8 h) DON-15-Glucuronide: 2.95 h total DON: 2.95 h	[11]
Factor for metabolic conversion (Fue) **	Fue for DON: 0.14 (0.03–0.42); RDF: 7.20 (CI 2.37–29.1) Fue for DON-15-Glucuronide: 0.37 (0.13–0.71); RDF: 2.67 (CI 1.4–7.96) Fue for total DON: 0.69 (0.14–0.97); RDF: 1.45 (CI 1.03–7.10)	[11] RDF: Reversed dosimetry factor
Type of derivation method selected ***	Option 2 as described in [3]: HBM-GV derivation based on a defined external toxicity reference value (TRV) Note: total urinary volume per 24 h used differs from German HBM Commission	Estimation by means of TK extrapolation.
Key study, author(s), year	[17]	
Species	Animal: mice	
Exposure route of study	Oral	
Study length	Chronic	
Exposure dose and conditions	0, 0.115, 0.661 and 1.520 mg/kg bw/day in males and 0, 0.098, 0.506 and 1.126 mg/kg bw/day in females for 2 years	
Critical endpoint	Reduced body weight gain	
Point of departure (POD) value	BMDL05: 0.11 mg DON/kg bw/day	
Extrapolations of the starting point	External TRV (group-TDI for DON, 3-acetyl-DON, 15-acetyl-DON and DON-3-Glucoside of 1 µg/kg bw/day) to HBM-GV (linked to urinary total DON by means of TK modelling).	
PBTK model for animal-human data extrapolation or intra-species extrapolation	[11]	
Assessment factors (AFs)	100 (from animal POD to external TRV taking into account inter- and intra-species variability into account)	
Rounded value of the HBM-GV	Total DON: 0.69 ug DON/kg bw/total 24 h ≡ 23 µg DON/L urine (CI: 5–33 µg/L) # DON-15-glucuronide (DON15GlcA): 0.60 ug DON15GlcA/kg bw/total 24 h ≡ 20 µg DON15GlcA/L urine (CI: 7–39 µg/L) #	# Based on a 24-h urine sample
Additional comments	Derivation of the HBM-GV was not based on a steady-state situation due to an elimination half-life that is shorter than the dosing interval	

* The half-lives of DON, DON-3-GlcA, and DON-15-GlcA were calculated based on the model and the calculated model parameter values described by Mengelers et al. (2019). These half-lives have been defined as the time value at which the cumulative excretion function of each metabolite has a value of 0.5 (50% of the total amount of DON or DON-glucuronide excreted). ** Fue is the inverse of the reversed dosimetry factor (RDF) as described by Mengelers et al. (2019). *** The equation of option 2 was used because it is applicable to this specific situation where 95% of the total DON is excreted within 12 h and not because a steady state has been reached. # Based on a 24-h urine sample.
